# The Prevalence of Metabolic Syndrome and Cardiovascular Risk Factors in Obese Children and Adolescents in Dalmatia: A Hospital Based Study

**DOI:** 10.1155/2016/1823561

**Published:** 2016-09-26

**Authors:** Marko Šimunović, Joško Božić, Lukrecija Milić, Ivana Unić, Veselin Škrabić

**Affiliations:** ^1^Department of Pediatrics, University Hospital Split, Spinčićeva 1, 21000 Split, Croatia; ^2^Department of Pathophysiology, University of Split, School of Medicine, Šoltanska 2, 21000 Split, Croatia; ^3^Department of Obstetrics and Gynecology, University Hospital Split, Spinčićeva 1, 21000 Split, Croatia

## Abstract

Obesity and metabolic syndrome (MS) are one of the biggest public health issues in child and adolescent population. To the best of the authors' knowledge, this hospital based study is the first report on the prevalence of MS in obese children and adolescents in Dalmatia, the Mediterranean part of Croatia. The objectives of this study were to determine the prevalence of individual cardiovascular risk factors and MS. Between January 2009 and June 2014, 201 obese subjects aged 6 to 18 were analyzed retrospectively from our Pediatric Endocrine Unit database. The subjects were then classified in two groups of obesity; subjects with BMI *z* score 2.0–3.0 were classified as moderately obese and subjects with BMI *z* score > 3.0 were classified as severely obese. The overall prevalence of MS using the modified IDF criteria was 30.3%. The most common component of MS in both groups was arterial hypertension, while impaired fasting glucose was the least common component of MS. Our finding of high prevalence of MS underlines the importance of early childhood obesity treatment.

## 1. Introduction

The prevalence of obesity in population of children and adolescents is still rising rapidly and it is one of the biggest public health issues in the world today [1–3]. Approximately today every fifth child and adolescent are obese in the US, while in Europe the prevalence of obesity ranges from 5% to 31% depending on reports from different countries [4, 5]. Therefore, obesity has become a matter of great concern because children and adolescents are the most vulnerable group and this period of life is critical for the development of a healthier future [6, 7]. Childhood obesity usually persists into adulthood, which may result in an increase in cardiovascular morbidity and mortality later in life [3, 7–9].

There is a wide opinion that the rising prevalence of obesity is connected with the growing number of early complications in children and adolescents, such as type 2 diabetes mellitus (T2D) and metabolic syndrome (MS) [1, 10, 11]. MS is defined as a cluster of cardiovascular risk factors: impaired glucose tolerance, dyslipidemia (elevated triglycerides and low high-density lipoprotein cholesterol (HDL)), hypertension, and abdominal obesity [12–14]. Unfortunately, there is a controversy regarding a uniform widely accepted definition of MS for children and adolescents [12, 15]. Approximately 40 different criteria are used to evaluate MS in children and adolescents, which makes it difficult to compare prevalence of MS between studies [2, 16]. Some authors point out that in the same group of children the prevalence of MS varies between 0 and 60% depending on the diagnostic criteria applied in research [16]. The explanation could lie in the fact that there is no consensus in literature on values of cut-off points for individual components of MS in children and adolescents [17]. Some pediatric studies use gender-specific percentiles from national reference data or study-specific distributions to determine thresholds for abnormalities of the MS components due to constant growth and developmental changes in childhood [18–20]. The International Diabetes Federation (IDF) in 2007 issued a unifying definition of MS in children and adolescents, and this progress enabled the comparison of different studies in the future [20]. Many studies are published on the prevalence and impact of the MS in the adult population, but there is limited information on the epidemiology of MS in children and adolescents, especially in Eastern Europe and the Mediterranean area [10, 17].

The aims of this study were to determine the effect of different degrees of obesity in children and adolescents on the individual cardiovascular risk factors and the prevalence of MS.

## 2. Materials and Methods 

The study protocol was previously reviewed and approved by the Ethics and Research Committee of the University Hospital Split and was conducted according to the ethical standards of the institution and the Declaration of Helsinki. The data was analyzed retrospectively from our Pediatric Endocrine Unit database. For all patients included in our study, we used the internal standardized protocol for the evaluation of children and adolescents with obesity.

### 2.1. Subjects

We observed 201 obese subjects aged 6 to 18 who were referred by their family practitioner or primary care pediatricians to our Pediatric Endocrine Unit for the evaluation of obesity in the period between January 2009 and June 2014. Exclusion criteria were obesity associated with genetic syndromes, diseases, and/or medications that alter blood pressure, glucose, and lipid metabolism.

### 2.2. Anthropometric Assessment

Body height and weight were measured using a calibrated scale (SECA Medical, Massachusetts, USA). The body mass index (BMI) was calculated as the weight in kilograms divided by the square of the height in meters. A detailed medical and family history was obtained. A meticulous physical examination was performed in search of abnormalities, such as acanthosis nigricans in the neck and axillary region. Pubertal development was assessed according to the Tanner staging system (I–V) [21, 22]. Tanner stage I was defined as prepubertal and Tanner stages II to V were defined as puberty [21, 22]. Blood pressure was measured according to the protocol recommended by the Update on the Task Force Report (1987) on High Blood Pressure in Children and Adolescents [23]. Blood pressure was measured three times while the subjects were seated, and the last two measurements were averaged for analysis [24].

### 2.3. Laboratory Assays

Blood sampling was performed after ≥12-hour overnight fast. Subjects underwent oral glucose tolerance test (OGTT) (1.75 g/kg glucose, with the maximum of 75 g); plasma insulin and blood glucose concentrations were measured at 0, 30, 60, 90, and 120 minutes. Plasma insulin was determined by the electrochemiluminescence immunoassay (ECLIA) method (Roche Diagnostics GmbH, Mannheim, Germany) and plasma glucose was measured using photometry with the hexokinase method (Abbott, Chicago, USA). Other laboratory assays were performed using routine laboratory methods.

### 2.4. Definitions

MS was defined according to the modified IDF criteria for children and adolescents [20]. Body proportions normally change during puberty and differences in waist circumference are difficult to interpret in children [25–28]. Therefore, we defined obesity on the basis of a threshold BMI *z* score of >2.0, adjusted for age and sex [29]. The subjects were then classified in two groups of obesity; subjects with BMI *z* score 2.0–3.0 were classified as moderately obese and subjects with BMI *z* score > 3.0 were classified as severely obese. The subjects in our study were diagnosed with MS if they had a BMI above the 97th percentile (BMI *z* score > 2.0) and two or more of the following criteria: triglyceride level ≥ 1.7 mmol/L, HDL ≤ 1.03 mmol/L, fasting glucose ≥ 5.6 mmol/L, and arterial hypertension (systolic blood pressure ≥ 130 mmHg and/or diastolic blood pressure ≥ 85 mmHg).

Impaired fasting glucose was defined as a fasting glucose value from 5.6 to 6.9 mmol/L, and impaired glucose tolerance (IGT) was defined as a glucose value after 120 minutes of OGTT from 7.8 to 11.0 mmol/L [30]. T2D was defined as a fasting glucose value ≥7.0 mmol/L and/or a glucose value after 120 minutes of OGTT ≥ 11.1 mmol/L [30]. The degree of insulin resistance was determined by the use of a homeostatic model assessment (HOMA-IR) [31]. HOMA-IR was calculated as the product of the fasting plasma insulin level (mIU/L) and fasting plasma glucose level (mmol/L), divided by 22.5 [31]. According to recent literature insulin resistance was defined as HOMA-IR score ≥ 3.16 and fasting insulin hyperinsulinemia was defined as concentration above 17 mIU/L [32, 33]. Insulin sensitivity was determined with widely used approximation Matsuda insulin sensitivity index (ISI) [34]. ISI was calculated as 10000/square root of [fasting glucose × fasting insulin] × [mean glucose × mean insulin during OGTT] [34].

### 2.5. Statistical Analysis

The data were analyzed using the statistical software MedCalc for Windows, version 11.5.1.0 (MedCalc Software, Mariakerke, Belgium). Continuous data were presented as means ± standard deviation, whereas categorical variables were presented as whole numbers and percentages. The Kolmogorov-Smirnov test was used to assess normality of data distribution. Means between the two groups were compared by the independent *t*-test. Categorical data were analyzed using Chi-square test. The statistical significance was set at *P* < 0.05.

## 3. Results

From a total of 201 children and adolescents, 110 (54.7%) subjects were moderately obese and 91 (45.3%) were severely obese, with a mean age for all subjects of 13.09 ± 2.71 years. Baseline anthropometric characteristics of the subjects enrolled in the study are presented in Table 1. There were no significant differences in anthropometric characteristics between these groups, except that the moderately obese subjects were significantly older than the severe obese subjects.

Comparisons between moderately and severely obese subjects regarding other clinical and biochemical characteristics as well as prevalence of individual risk factors are presented in Tables 2 and 3. Prevalence of arterial hypertension, insulin resistance, and hyperinsulinemia was significantly higher in the group of severely obese subjects. The most common component of MS in both groups of obese children and adolescents was arterial hypertension, while impaired fasting glucose was the least common component of MS.

The overall prevalence of MS in our study group using the modified IDF criteria was 30.3%, with the prevalence of 24.5% in the group of moderately obese and 37.4% in severely obese subjects. The majority of children and adolescents in our study had one or two components of MS. The prevalence of individual components of MS between moderately and severely obese groups was as follows: one component (34.6% versus 29.6%, *P* = 0.462), two components (40.9% versus 33.0%, *P* = 0.247), three components (20.0% versus 25.3%, *P* = 0.372), four components (4.5% versus 11.0%, *P* = 0.084), and five components (0% versus 1.1%, *P* = 0.270).

Only one of the obese children and adolescents had T2D. Comparisons of baseline anthropometric and biochemical characteristics of subjects with and without MS are presented in Table 4. Subjects with MS had higher body mass and height, were significantly older, and had significantly higher mean values of BMI, glucose after 120 minutes in OGTT, and Matsuda index.

## 4. Discussion 

This study reports very high prevalence of MS in obese children and adolescents. Our study subjects were classified in two groups according to degrees of obesity and higher prevalence of MS and significantly more individual components of MS were found in the group of severely obese subjects. This finding underlines the importance of recognizing degrees of obesity in children and adolescents, which could potentially influence different rates of developing cardiovascular risk factors [35]. After an extensive review of available literature we would like to point out that our hospital based study is the first report on the characteristics and prevalence of MS in obese children and adolescents in Dalmatia, the Mediterranean part of Croatia.

In comparison with other studies our results showed high prevalence of MS compared to other Mediterranean countries, which varies from 7.7% in the population of obese Greece children and 8.9% in the population of obese Portuguese adolescents to 23.3% in the population of extremely obese Italian adolescents—all using the IDF criteria [8, 14, 36]. Very similar prevalence of MS in comparison with our study was found in group of extremely obese German adolescents, but with a higher average BMI *z* score [8]. This difference in the prevalence of MS could be partially explained by the fact that Croatian population of children and adolescents in the past 20 years went through extensive sociopolitical changes in the postwar period which have extremely influenced early growth and development, nutritional habits, physical activity, and cultural environment [37, 38]. These transitional changes are well known leading factors that increase the prevalence of obesity and other cardiovascular risk factors [37, 38]. Other factors may influence the prevalence of MS such as different mean age of subjects, as well as differences in degrees of obesity of subjects (higher or lower values of mean BMI and BMI *z* score). Additionally, several publications emphasize the importance of Mediterranean diet in reducing of prevalence of MS and individual components of MS [39, 40]. The aforementioned points to the complexity of comparisons of MS prevalence and influence of other multiple factors on prevalence of MS.

The IDF criteria for children and adolescents use waist circumference for defining obesity [20]. In our study the IDF criteria were modified and BMI *z* score was used for defining obesity. A BMI *z* score is a widely accepted criterion for defining degree of obesity in children and adolescents, because there is lack of a well-accepted international classification for waist circumference with age and gender specific cut-off points [27, 28]. Some authors point out that the BMI and BMI *z* score are superior in the prediction of insulin resistance and MS in pediatric population when compared with other parameters [28, 41].

The most used criteria for pediatric population for diagnosis of MS are IDF criteria [1, 8, 9]. The prevalence of MS strongly depends on different approaches to define pediatric MS [16]. Currently, there are several published studies that compared the prevalence of MS using different definitions. In a large cohort study Xu et al. reported the prevalence of MS in a sample of nearly 9,000 school age Chinese children, which ranged depending on the criteria from 0.8% using IDF criteria to 11.6% using Ferranti et al. criteria [42]. Similar results were found in a study of five European countries on a sample of over 1,200 obese children in which the lowest prevalence of MS was 16.4% using the IDF criteria, and the highest 35.7% using Ferranti et al. criteria [43]. Unfortunately it is very difficult to assess which criteria are optimal, due to very heterogeneity of the studied groups.

Since there are different approaches in defining MS in pediatric population we would like to underline the importance of percentiles adjusted for age and gender. The importance of percentile curves was verified in the publication of Martino et al., which analyzed cardiovascular risk factors and formed percentiles adjusted for age and gender in large sample of more than 1,500 children aged from 6 to 14 years in the general population of children from Southern Italy [44]. Interestingly, hypertension was defined as the value >95th percentile from regional sample and that values were significantly lower in comparison with relatively high IDF cut-off points [44]. In contrast, the same group of authors when they used the percentile curves for the definition of MS in the same population reported a relatively low prevalence of MS less than 5% [45]. These results indicate a major difference in the prevalence of MS depending on geographic region and ethnic origin. Perhaps IDF cut-off points are easier to use in everyday practice, but we and other authors recommend the use of percentiles especially in early childhood, because percentiles take into account the physiological changes that occur during growth and development [7]. Long-term surveys of the general population are necessary for better understanding and definition of national standardized cut-off points for the components of MS for different stages of childhood.

Insulin resistance is the first step in the development of abnormalities of glucose homeostasis found in obesity [46]. In our study we have found relatively high prevalence of insulin resistance in both groups of obese subjects, which is extremely difficult to compare with other studies since other authors used different cut-off points for HOMA-IR (1.8, 2.5, 3.2, to 4) depending on the population [32]. Kurtoglu et al. reported similar incidence of insulin resistance which was estimated with multiple models (HOMA-IR, ISI,…) in both groups with MS and without MS using the IDF criteria [47]. There was no difference in HOMA-IR in our subjects with MS and those without MS. In contrast, in our study ISI was significantly lower in the group with MS. HOMA-IR is a commonly used model due to its simplicity, but ISI is a more complex assessment of insulin resistance and takes into account not only the fasting state, but concentrations of insulin and glucose during the OGGT. ISI should be taken into consideration for future assessments of insulin resistance in pediatric population; however, due to the financial nature unambiguous clinical criteria for everyday use should be defined.

Our study has also found a low rate of impairment of glucose metabolism in moderately and severely obese subjects. These findings are in contrast with the rising incidence of T2D in obese children and adolescents worldwide [48]. IGT rates are lower in our study than estimates of IGT rates in the US study (21–25%), but similar to the European study (7.5%) [48, 49]. It is likely that these variations reflect differences between clinical and ethnic populations. Our findings may indicate a wide range of abnormalities of glucose homeostasis associated with obesity in childhood. Potentially early weight loss can lead to reduced prevalence of impairment of glucose metabolism and juvenile T2D [50].

This study has some limitations. Firstly, the data from these clinical samples may not be representative of the general Croatian pediatric population. However, this was a considerable regional sample that represents the population of major importance for physicians and includes a significant number of obese children and adolescents. Secondly, modifications of the IDF standard MS criteria may also have affected our results. Additional studies are needed which would include control group of nonobese subjects which will be beneficial to future research and contributed to a better understanding of MS in children and adolescents.

In conclusion, we confirm that the development of cardiovascular risk factors and MS has a strong connection with childhood obesity. Our results show a high prevalence of MS in the obese child and adolescent population, thus indicating the possibility of early complications in adulthood. Therefore, the prevention and treatment of early childhood obesity and MS play the most important role in reducing the risk of cardiovascular disease in adulthood. Furthermore, we must point out once again the importance of standardization of diagnostic criteria of MS in pediatric population.

## Figures and Tables

**Table 1:** Baseline anthropometric characteristics of subjects enrolled in the study^*∗*^.

Parameter	Moderately obese (*N* = 110)	Severely obese (*N* = 91)	*P*
Gender, *N* (%)			
Male	54 (49.1)	50 (54.9)	0.408
Female	56 (50.9)	41 (55.1)	
Age (yr)	13.44 ± 2.41^†^	12.66 ± 2.96	0.047
Height (cm)	163.80 ± 12.84	162.03 ± 15.97	0.403
Weight (kg)	79.07 ± 15.90	92.88 ± 25.25	<0.001
BMI (kg/m^2^)	29.01 ± 2.54	34.62 ± 4.67	<0.001
BMI *z* score	2.64 ± 0.34	3.43 ± 0.31	<0.001
Pubertal status, *N* (%)			
Prepubertal	12 (10.9)	17 (18.7)	0.157
Pubertal	98 (89.1)	74 (81.3)	

^*∗*^Obese subjects with a body mass index (BMI) converted to a *z* score of 2.0 to 3.0 were classified as moderately obese, and subjects with a *z* score > 3.0 were classified as severely obese. ^†^Values are presented as mean ± SD, except where unless otherwise stated.

**Table 2:** Biochemical and clinical characteristics of subjects enrolled in the study.

Parameter	Moderately obese (*N* = 110)	Severely obese (*N* = 91)	*P*
Acanthosis, *N* (%)	52 (47.3)	54 (59.3)	0.088
Total cholesterol (mmol/L)	4.37 ± 0.88^†^	4.44 ± 0.75	0.519
Triglycerides (mmol/L)	1.37 ± 0.75	1.42 ± 0.70	0.653
HDL^*∗*^ (mmol/L)	1.27 ± 0.41	1.24 ± 0.31	0.610
LDL^†^ (mmol/L)	2.46 ± 0.83	2.52 ± 0.70	0.628
Fasting glucose (mmol/L)	4.86 ± 0.55	4.79 ± 0.51	0.402
Glucose 120 min (mmol/L)	6.14 ± 1.15	6.11 ± 1.28	0.871
Fasting insulin (mIU/L)	19.97 ± 13.71	24.28 ± 15.01	0.037
Insulin 120 min (mIU/L)	77.18 ± 75.50	86.74 ± 72.34	0.364
HOMA-IR^‡^	4.35 ± 3.22	5.21 ± 3.21	0.063
ISI^§^	3.61 ± 2.37	3.09 ± 2.76	0.163
SBP^*‖*^ (mmHg)	124.50 ± 14.52	129.91 ± 16.96	0.018
DBP^¶^ (mmHg)	78.78 ± 9.09	82.90 ± 10.45	0.004

^*∗*^HDL: high density lipoproteins, ^†^LDL: low density lipoproteins, ^‡^HOMA-IR: homeostatic model assessment insulin resistance, ^§^ISI: Matsuda index, ^*‖*^SBP: systolic blood pressure, and ^¶^DBP: diastolic blood pressure.

**Table 3:** Prevalence of individual risk factors in moderately and severely obese subjects.

Parameter	Moderately obese (*N* = 110)	Severely obese (*N* = 91)	*P*
	*N* (%)	*N* (%)	
Hypertriglyceridemia	28 (25.5)	27 (29.7)	0.505
Low HDL^*∗*^	29 (26.4)	28 (30.8)	0.490
Impaired fasting glucose	9 (8.2)	8 (8.8)	0.877
Arterial hypertension	38 (34.5)	47 (51.6)	0.014
Insulin resistance	61 (55.5)	66 (72.5)	0.013
Hyperinsulinemia	52 (47.3)	61 (67.0)	0.005
Impaired glucose tolerance	10 (9.1)	4 (4.4)	0.334

^*∗*^HDL: high-density lipoproteins.

**Table 4:** Anthropometric and biochemical characteristics of children with and without MS.

Parameter	Without MS (*N* = 140)	With MS (*N* = 61)	*P*
Age (yr)	12.74 ± 2.65	13.87 ± 2.66	0.007
Gender, *N* (%)			
Male	70 (50.0)	34 (55.7)	0.454
Female	70 (50.0)	27 (44.3)	
Height (cm)	161.20 ± 13.81	167.10 ± 14.79	0.010
Weight (kg)	81.80 ± 20.72	93.41 ± 21.99	<0.001
BMI^*∗*^ (kg/m^2^)	30.99 ± 4.46	32.85 ± 4.65	0.010
BMI *z* score	2.97 ± 0.53	3.07 ± 0.46	0.187
Acanthosis	77 (55.0)	29 (47.5)	0.330
Total cholesterol (mmol/L)	4.43 ± 0.83	4.35 ± 0.80	0.521
Triglycerides (mmol/L)	1.17 ± 0.48	1.90 ± 0.93	<0.001
HDL^†^ (mmol/L)	1.35 ± 0.37	1.04 ± 0.24	<0.001
LDL^‡^ (mmol/L)	2.47 ± 0.79	2.51 ± 0.74	0.743
Fasting glucose (mmol/L)	4.789 ± 0.46	4.93 ± 0.65	0.132
Glucose 120 min (mmol/L)	6.00 ± 1.16	6.43 ± 1.26	0.027
Fasting insulin (mIU/L)	21.71 ± 14.94	22.42 ± 13.32	0.738
Insulin 120 min (mIU/L)	78.05 ± 77.93	89.45 ± 64.27	0.285
HOMA-IR^§^	4.64 ± 3.23	4.97 ± 3.27	0.523
ISI^*‖*^	3.59 ± 2.78	2.87 ± 1.89	0.036
SBP^¶^ (mmHg)	123.50 ± 14.37	134.80 ± 16.45	<0.001
DBP^*∗∗*^ (mmHg)	79.00 ± 9.42	84.43 ± 10.12	<0.001

^*∗*^BMI: body mass index, ^†^HDL: high-density lipoproteins, ^‡^LDL: low density lipoproteins, ^§^HOMA-IR: homeostatic model assessment insulin resistance, ^*‖*^ISI: Matsuda index, ^¶^SBP: systolic blood pressure, and ^*∗∗*^DBP: diastolic blood pressure.
